# Suicide and Suicidal Ideation in Medical Students: A Systematic Review

**DOI:** 10.7759/cureus.65246

**Published:** 2024-07-24

**Authors:** Azfar Mateen, Visesh Kumar, Ajay K Singh, Berendra Yadav, Mala Mahto, Sumit Mahato

**Affiliations:** 1 Forensic Medicine, Mahamaya Rajkiya Allopathic Medical College (MRAMC), Ambedkarnagar, IND; 2 Biochemistry, Mahamaya Rajkiya Allopathic Medical College (MRAMC), Ambedkarnagar, IND; 3 Physiology, Mahamaya Rajkiya Allopathic Medical College (MRAMC), Ambedkarnagar, IND; 4 Biochemistry, All India Institute of Medical Sciences (AIIMS), Patna, IND; 5 Pharmacology, All India Institute of Medical Sciences (AIIMS), Deoghar, IND

**Keywords:** depression, marital discord, suicidal ideation, suicide, medical student

## Abstract

The incidence of suicide and its ideation among medical students is increasingly recognized as a significant issue, influenced by the demanding nature of medical education and the various associated psychological stressors. This systematic review aims to elucidate the risk factors and prevalence of suicide and suicidal ideation within this group, with a focus on the implications for mental health interventions. Following the Preferred Reporting Items for Systematic Reviews and Meta-Analyses (PRISMA) guidelines, this review analyzed 11 studies selected from major databases such as Scopus-Elsevier, Google Scholar, PubMed, and others, focusing on publications between 2015 and 2023. Studies were predominantly observational and cross-sectional, examining suicide rates and suicidal ideation among medical students. The review found that medical students experience higher rates of suicide and suicidal ideation compared to the general population, with significant stressors including academic pressure, personal relationship challenges, and professional expectations. Notably, female respondents displayed a higher prevalence of suicidal ideation than males. Key warning signs identified include changes in mood or behavior and previous attempts of suicide. Despite varying findings regarding the role of medical training duration on suicidal ideation, all studies highlight the critical need for targeted mental health support. The findings underscore the urgent need for integrated mental health services and the incorporation of mental health education within the medical curriculum. A multidimensional approach involving enhancements to both curricular and support structures is crucial for mitigating the risk of suicide among medical students. Future research should focus on developing and evaluating interventions to reduce educational stress and promote psychological well-being in medical educational settings.

## Introduction and background

The act of suicide is heartbreaking and has a profound impact on society. Suicide involves intentionally causing one's own death [1]. The causes of suicide stem from a complex interplay of environmental, social, biological, and cultural factors. The term "ideation of suicide" refers to thoughts about, considerations of, or planning to end one's life. It is crucial to identify risk factors to both prevent suicide and promote mental well-being [2]. Recent literature shows a gradual increase in the prevalence of suicide and suicidal ideation among medical students [3]. Compared to the general population, medical students exhibit more mental health issues, likely due to stressful educational environments and high expectations for achievement [4,5]. These factors can significantly affect a student's life, either positively or negatively. While mild stress can enhance creativity and performance, severe stress detrimentally affects students' physical and mental well-being. Prolonged psychological stress may impair a student's learning process, cognitive function, and academic achievements [6]. Furthermore, increased stress levels place medical students at a heightened risk of developing suicidal ideation [7].

The purpose of this systematic review is to explore the causes of suicide and to mitigate psychiatric morbidity among medical students, which may manifest as depression or suicidal thoughts. Understanding these factors is essential to enhance the efficacy of intervention strategies and implement effective prevention programs for medical students, aiming to reduce future incidents of suicide among physicians.

## Review

Search strategy and selection criteria

This study adhered to the guidelines set forth by the Preferred Reporting Items for Systematic Reviews and Meta-Analyses (PRISMA) (see Appendices) [8]. We utilized several electronic databases for our search, including Scopus-Elsevier, Google Scholar, PubMed, Cochrane Central Register of Controlled Trials, and the Clinical Trials Registry-India. Searches were limited to documents published in English from 2015 to 2023 and were further categorized by geographical focus, specifically India and the rest of the world. Our search terms were based on the Medical Subject Headings and included "suicide," "students," and "India," along with keywords like psychiatric morbidity, medical students, physician, depression, anxiety, stress, addiction, suicide ideation, and suicide. We employed a combination of these nine keywords using "AND" and "OR" as Boolean operators. Additionally, we examined the reference lists of all relevant articles to identify further studies and adhered to established guidelines for systematic review preparation [9,10]. All publications linked in the related articles function in PubMed were also screened.

Study inclusion and exclusion criteria

The inclusion criteria for this review were strictly followed: studies must have been published between 2015 and 2023, in English, involving medical students, and primarily observational cross-sectional studies. Exclusion criteria included unrelated research, studies lacking sufficient data, duplicates, interventional studies, and those concerning undergraduate or postgraduate students not pursuing a Bachelor of Medicine, Bachelor of Surgery, or MD/MS degree, respectively.

**Figure 1:**
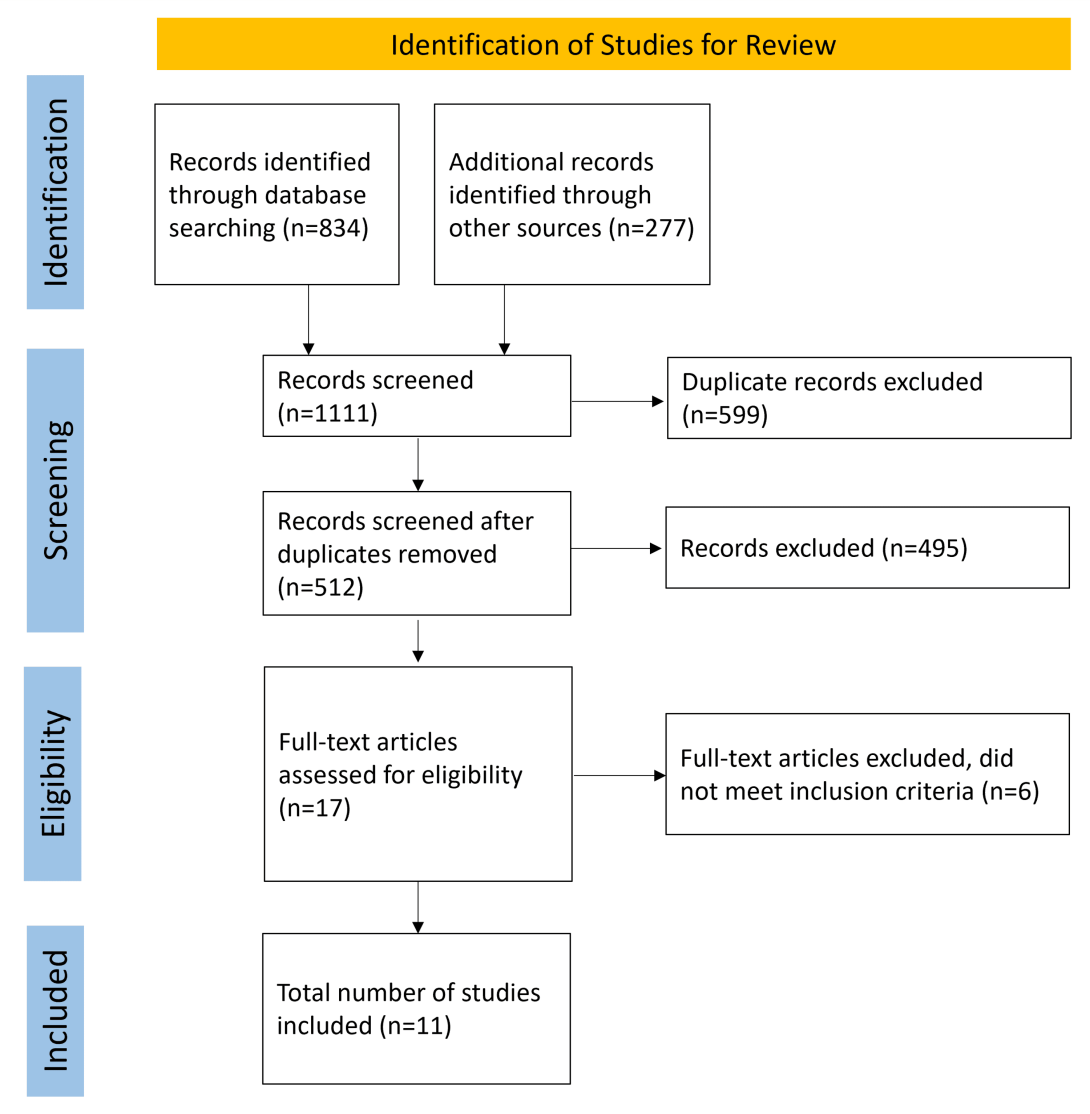
Screening and inclusion of articles using the PRISMA flowchart PRISMA: Preferred Reporting Items for Systematic Reviews and Meta-Analyses

After applying these criteria, 1,111 studies were initially identified. Through a two-phase review process involving an assessment of abstracts and then full texts, the selection was narrowed to 11 studies that met all requirements (Figure 1) [11-21]. Three of the authors AM, AKS, and SKM performed the literature search independently to identify the studies for inclusion in the present review. VK, BY, and MM then extracted the pre-specified data from the studies. Any disagreements were resolved by consensus. These studies, which included both male and female participants, provided data on suicide and ideation of suicide among medical students (Table 1). The excluded studies involved those published before 2017, perspectives, letters to editors, and those deemed irrelevant.

**Table 1:** Review of studies on suicide and suicidal ideation among medical students NMC: National Medical Commission; MCI: Medical Council of India; MBBS: Bachelor of Medicine, Bachelor of Surgery

Author, year	Study population	Data collection method	Sample size, study design	Suicide/suicide ideation	Respondent location	Conclusions	Remarks
Chahal et al., 2022 [11]	MBBS students, residents, physicians	Online news portal and Google database	N=358, record-based retrospective study	Suicide	India	Academic stress is a key reason for suicide among students and residents, while marital discord is significant among physicians. The most common method of suicide was hanging; poisoning was prevalent among residents and physicians.	Advocates for mandatory reporting of suicide in medical professionals to designated authorities.
Chaudhary et al., 2019 [12]	Medical students	News articles and web/electronic databases	N=196, record-based retrospective study	Suicide	India	Reporting of suicide has quadrupled over the last decade, with a significant proportion originating from 13 medical colleges.	Calls for mandatory reporting and record-keeping improvements by the NMC (formerly MCI).
Kishor et al., 2021 [13]	MBBS student	Suicide report from an online platform	N=8, record-based retrospective study	Suicide	India	Suicide is notably high among young Indian female doctors, often linked to unaddressed psychiatric issues.	Suggests the need for comprehensive suicide prevention policies at medical institutions.
Nebhinani et al., 2020 [14]	MBBS students	Proforma	N=158, cross-sectional study	Suicide ideation	India	Students' suicide ideation is influenced by personal, coaching, and familial factors.	Recommends qualitative studies to inform policymaking for suicide prevention.
Ram et al., 2017 [15]	Medical students	Questionnaire-based	N=151, observational study	Suicide ideation	India	Depression and suicide literacy are prevalent among healthcare professional students, and suicidal tendency was associated with depression literacy.	Suggests incorporating mental health education into the MBBS curriculum to address depression and suicide.
Nesan et al., 2020 [16]	Undergraduate medical students	Questionnaire-based	N=415, cross-sectional	Suicide ideation	India	Higher suicidal ideation was observed in students experiencing depression and isolation.	Advocates for a multi-pronged approach involving counseling and mental health services.
Nebhinani et al., 2020 [17]	MBBS student	Semi-structured proforma	N=243, prospective study design	Suicide ideation	India	The majority of students show a positive attitude towards suicide prevention.	Proposes including suicide prevention strategies in the medical curriculum.
Nakhostin-Ansari et al., 2022 [18]	MBBS student	Questionnaire	N=133, cross-sectional study	Suicide ideation	Iran	High prevalence of suicidal ideation, particularly among students with family psychiatric histories and personal challenges.	Recommends addiction interventions and curriculum adjustments to reduce stress and increase satisfaction.
Goyal et al., 2012 [19]	MBBS student	Questionnaire	N=200, cross-sectional study	Suicide ideation	India	High suicidal ideation rates can be addressed through enhanced recreational activities and curriculum restructuring.	Emphasizes the need for counseling programs for medical students.
Desalegn et al., 2020 [20]	MBBS student	Questionnaire	N=423, cross-sectional study	Suicide ideation	Ethiopia	Although suicide ideation is low, attempts are high, influenced by depression, substance use, and inadequate social support.	Urges government development of policies to address suicide ideation among medical students.
Desai et al., 2021 [21]	MBBS student	Questionnaire	N=731, record-based retrospective study	Suicide ideation	India	A notable percentage of students seriously considered taking their lives in the past month.	Supports the use of web-based surveys to gauge suicidal ideation prevalence among students.

Analysis of the included studies on suicide

The analysis revealed that most participants recognized a stressful environment as a primary trigger for suicidal ideation or suicide. Three studies provided quantitative data on suicide rates among medical students, residents, and physicians, showing variations in gender distribution. Chahal et al. reported a higher male-to-female ratio of suicide except in residents, where females predominated [11]. Chaudhary et al. observed a similar trend but did not differentiate between residents and others [12], whereas Kishor et al. found a higher female-to-male ratio among doctors [13]. Hanging was the most common means of suicide in all three studies, followed by poison/drug use [11-13]. In terms of specialties, anesthesiology had the highest rates of suicide, followed by obstetrics according to Chahal et al. [11], pediatrics and general surgery in Chaudhary et al. [12], and dermatology in Kishor et al. [13].

The common causes of suicide included academic pressures among medical students (45.2%) and residents (23.1%), with marital problems being the most significant factor among physicians (26.7%). Mental health issues were the next most frequent cause among medical students (24%) and physicians (20%), while harassment was a significant concern among residents (20.5%) [11-13].

Analysis of the included studies on suicide ideation

Eight studies reviewed suicidal ideation, with six originating from India and one each from Iran and Ethiopia [14-21]. Five of these studies reported a higher number of male participants [14,17-20], while three had predominately female participants [15,16,21]. Notably, four studies found that female suicide ideation was more prevalent. For instance, Nesan et al. studied 415 participants and reported percentages, noting that 87.5% experienced "mild" suicidal ideations, distributed between 49.6% of women and 37.8% of men [16]. Moreover, 8.9% had "moderate" (3.9% men and 5.1% women), and 3.6% had "severe" suicidal ideations (1.4% men and 2.2% women) [16].

Goyal et al. reported an overall prevalence of 53.6% (n=142) for suicidal ideation among medical students, with 2.7% (n=3) having attempted suicide at least once [19]. The gender-based prevalence showed significant differences, with 45.6% of men (n=63) and 62.2% of women (n=79) exhibiting suicidal ideation [19]. Multivariate analysis by Desalegn et al. indicated that women were more than five times as likely to exhibit suicide ideation compared to men [20]. Desai et al. found that female students were nine times more likely to have suicidal ideation than male students [21]. Conversely, Ansari et al. found no significant gender differences in the prevalence of suicidal ideation among 133 medical students, with 45 students showing ideation at the time of the study [18].

Further, Desai et al. [21] noted that in the past month, 66 students (13%) felt life was not worth living, 32 (6%) experienced death wish, and 22 (4%) considered taking their life without intent to act on these thoughts. Notably, five students (1%) seriously considered taking their life, and two had attempted suicide within the same timeframe. Besides these two, an additional 21 students (4%) had a history of attempting suicide. Goyal et al. [19] found a significantly higher rate of suicidal ideation among students exhibiting impulsive behaviors in challenging situations: 70.9% compared to 30.7% in those who did not exhibit such behaviors. A noteworthy 57% of students displayed impulsive behaviors, with 78% of those having suicidal ideation feeling overwhelmed, in contrast to 4.5% who did not report such feelings.

Nesan et al. [16] detailed that 4.1% of female participants often had suicidal thoughts, compared to 2.4% of males. Additionally, 3.6% of male respondents expressed readiness to commit suicide, against 2.2% of female respondents. About 17.1% of women versus 11.3% of men expressed indifference to living, and 8.4% of women, compared to 4.3% of men, reported wishing they were dead. Within the past year, 13 participants harbored intense suicidal thoughts; three were prepared to commit suicide if means were available, three had completed preparations for suicide, 10 had finished suicide notes, and seven had already attempted suicide.

Discussion

Our study aimed to systematically overview all risk factors associated with suicide ideation and suicide. Physicians worldwide often operate under stressful conditions and must make critical decisions under intense pressure. These pressures have only increased with escalating expectations from patients and their families. Recent observations indicate that the rates of psychiatric disorders, particularly suicide, are significantly higher among doctors compared to the general population [22]. The literature also highlights that medical students are particularly vulnerable to suicide. Despite India having the world's largest medical education system, the country lacks definitive, structured working hours and conditions. High-risk groups include males, those experiencing academic stress, harassment, or bullying, and those struggling with personal relationships. Individuals with psychological comorbidities such as depression are also at increased risk. Notably, key warning signs observed in those who committed suicide include changes in mood or behavior, academic stress, and previous suicide attempts. Past studies have suggested that awareness of mental health symptoms, their identification, and the availability of targeted mental health services can mitigate suicide risks among medical students [23]. Our review comprises 11 studies, nine from India, one from Iran, and one from Ethiopia, illustrating that suicidal ideation is a global issue.

Our systematic review indicates an association between suicidal ideation and gender, with females more frequently experiencing suicidal ideation. This contrasts with a study by Desai et al., which found an inconsistent association between suicidal ideation and gender [21]. Our findings were also inconclusive regarding the influence of medical college year on the prevalence of suicidal ideation, leaving it unclear whether the prevalence increases or decreases over time in medical school. In India, medical students often prefer informal consultations and self-diagnosis due to privacy concerns. Additionally, students with psychiatric conditions perceive greater barriers to seeking mental healthcare compared to their peers without such conditions [24].

To prevent suicide and suicide ideation, measures focus on reducing academic stress. Strategies include improving the curriculum, providing opportunities for recreational activities, and incorporating mentor-mentee programs, all of which may enhance satisfaction with their field. Early screening and intervention for depression are also crucial for preventing suicide. Medical students and physicians should be educated about suicide warning signs to help them recognize these signs in themselves and their colleagues. Our review suggests that mandatory reporting of suicide incidents among medical students and physicians to designated authorities could provide crucial data for policymaking aimed at prevention. A multidimensional approach involving mental health services, curriculum support programs, and counseling centers is essential for the prevention of suicide.

Limitations

While this review provides important insights, it is limited by several factors. First, by including only articles published in English, we potentially missed significant studies published in other languages that could offer additional perspectives, especially relevant in non-English-speaking countries. Furthermore, our exclusion of qualitative studies may limit the depth of understanding regarding personal experiences and systemic issues that contribute to suicide. Additionally, the absence of a meta-analysis prevents us from quantifying effects and may reduce the generalizability of our findings.

## Conclusions

This systematic review highlights the complex interplay of stressors contributing to suicide and suicidal ideation among medical students. Our findings emphasize the need for a comprehensive approach to address mental health issues within the medical education system. Specifically, the association between increased stress and suicide ideation underlines the urgency of implementing targeted mental health interventions and supportive environments for medical students. Moreover, our study underscores the importance of including mental health education within the medical curriculum and ensuring access to mental health services, which could significantly mitigate risks. The implications for the medical community are clear: there is a critical need to reform educational and clinical practices to better support the mental well-being of future physicians. Further research should focus on developing and testing interventions that can effectively reduce stress and improve mental health outcomes among medical students, emphasizing inclusivity and accessibility to accommodate diverse student populations.

## Appendices

**Table 2:** PRISMA 2009 checklist PICOS: participants, interventions, comparisons, outcomes, and study; PRISMA: Preferred Reporting Items for Systematic Reviews and Meta-Analyses Reference: [8]

Section/topic	#	Checklist item	Reported on page #
Title			
Title	1	Identify the report as a systematic review, meta-analysis, or both.	1
Abstract			
Structured summary	2	Provide a structured summary including, as applicable, background; objectives; data sources; study eligibility criteria, participants, and interventions; study appraisal and synthesis methods; results; limitations; conclusions and implications of key findings; systematic review registration number.	1
Introduction			
Rationale	3	Describe the rationale for the review in the context of what is already known.	1
Objectives	4	Provide an explicit statement of questions being addressed with reference to PICOS design.	1
Methods			
Protocol and registration	5	Indicate if a review protocol exists and if and where it can be accessed (e.g., web address), and, if available, provide registration information including registration number.	N/A
Eligibility criteria	6	Specify study characteristics (e.g., PICOS, length of follow-up) and report characteristics (e.g., years considered, language, publication status) used as criteria for eligibility, giving rationale.	2
Information sources	7	Describe all information sources (e.g., databases with dates of coverage, contact with study authors to identify additional studies) in the search and date last searched.	2
Search	8	Present a full electronic search strategy for at least one database, including any limits used, such that it could be repeated.	2
Study selection	9	State the process for selecting studies (i.e., screening, eligibility, included in the systematic review, and, if applicable, included in the meta-analysis).	2
Data collection process	10	Describe the method of data extraction from reports (e.g., piloted forms, independently, in duplicate) and any processes for obtaining and confirming data from investigators.	2
Data items	11	List and define all variables for which data were sought (e.g., PICOS, funding sources) and any assumptions and simplifications made.	2
Risk of bias in individual studies	12	Describe methods used for assessing the risk of bias of individual studies (including specification of whether this was done at the study or outcome level) and how this information is to be used in any data synthesis.	N/A
Summary measures	13	State the principal summary measures (e.g., risk ratio, difference in means).	N/A
Synthesis of results	14	Describe the methods of handling data and combining results of studies, if done, including measures of consistency (e.g., I^2^) for each meta-analysis.	N/A
Risk of bias across studies	15	Specify any assessment of risk of bias that may affect the cumulative evidence (e.g., publication bias, selective reporting within studies).	N/A
Additional analyses	16	Describe methods of additional analyses (e.g., sensitivity or subgroup analyses, meta-regression), if done, indicating which were pre-specified.	N/A
Results			
Study selection	17	Give the number of studies screened, assessed for eligibility, and included in the review, with reasons for exclusions at each stage, ideally with a flow diagram.	2 (Figure 1)
Study characteristics	18	For each study, present characteristics for which data were extracted (e.g., study size, PICOS, follow-up period) and provide the citations.	3 (Table 1)
Risk of bias within studies	19	Present data on the risk of bias of each study and, if available, any outcome level assessment (see item 12).	N/A
Results of individual studies	20	For all outcomes considered (benefits or harms), present, for each study, (a) simple summary data for each intervention group and (b) effect estimates and confidence intervals, ideally with a forest plot.	3 (Table 1)
Synthesis of results	21	Present results of each meta-analysis done, including confidence intervals and measures of consistency.	N/A
Risk of bias across studies	22	Present results of any assessment of risk of bias across studies (see item 15).	N/A
Additional analysis	23	Give results of additional analyses, if done (e.g., sensitivity or subgroup analyses, meta-regression (see item 16)).	N/A
Discussion			
Summary of evidence	24	Summarize the main findings including the strength of evidence for each main outcome; consider their relevance to key groups (e.g., healthcare providers, users, and policymakers).	4
Limitations	25	Discuss limitations at the study and outcome level (e.g., risk of bias) and at the review level (e.g., incomplete retrieval of identified research, reporting bias).	5
Conclusions	26	Provide a general interpretation of the results in the context of other evidence and implications for future research.	5
Funding			
Funding	27	Describe sources of funding for the systematic review and other support (e.g., supply of data) and the role of funders for the systematic review.	N/A
